# Case Report: Multivessel Coronary Disease Assessment with SPECT ^99m^Tc-Sestamibi and Rubidium-82 PET/CT

**DOI:** 10.5935/abc.20160198

**Published:** 2017-01

**Authors:** Bruno Gomes Padilha, Daniela Sabino, Maria Clementina Giorgi, José Soares Jr., Marisa Izaki, José Claudio Meneghetti

**Affiliations:** Serviço de Medicina Nuclear e Imagem Molecular do Instituto do Coração - Hospital das Clínicas - Faculdade de Medicina - Universidade de São Paulo (USP), São Paulo, SP − Brazil

**Keywords:** Coronary Artery Disease, Cardiac Catheterization, Myocardial Perfusion Imaging, Rubidium-82, Technetium Tc 99m Sestamibi, Radionuclide Imaging

## Introduction

Coronary angiography (CAG) is the standard diagnostic method for detection of coronary artery disease (CAD). However, it is often necessary to evaluate the expression of a coronary obstruction in relation to myocardial perfusion, before defining the best patient management.

Myocardial perfusion scintigraphy with technetium-99m-Sestamibi (^99m^Tc-sestamibi) allows early detection and evaluation of disease extension and cardiovascular risk in patients with suspected or established CAD, helping in decision-making regarding the start and type of therapy to be implemented.^1^ This method has been widely used, but shows difficulties in some situations such as balanced multivessel disease, in which the proportional flow distribution in the myocardial regions can hinder ischemia detection. In such cases, additional assessment data, such as evaluation of contractility, decrease in left ventricular ejection fraction (LVEF) under stress, electrocardiographic alterations or symptoms during stress, dilation of the left ventricle (LV) cavity under stress can provide evidence of ischemia, indicating further diagnostic investigation.

Noninvasive imaging using Positron-Emission Computed Tomography (PET-CT) allows the acquisition of myocardial perfusion imaging with better quality than conventional equipment, in addition to estimating quantitative measures of myocardial blood flow at rest and under stress, as well as of coronary reserve.

**Figure 1:**
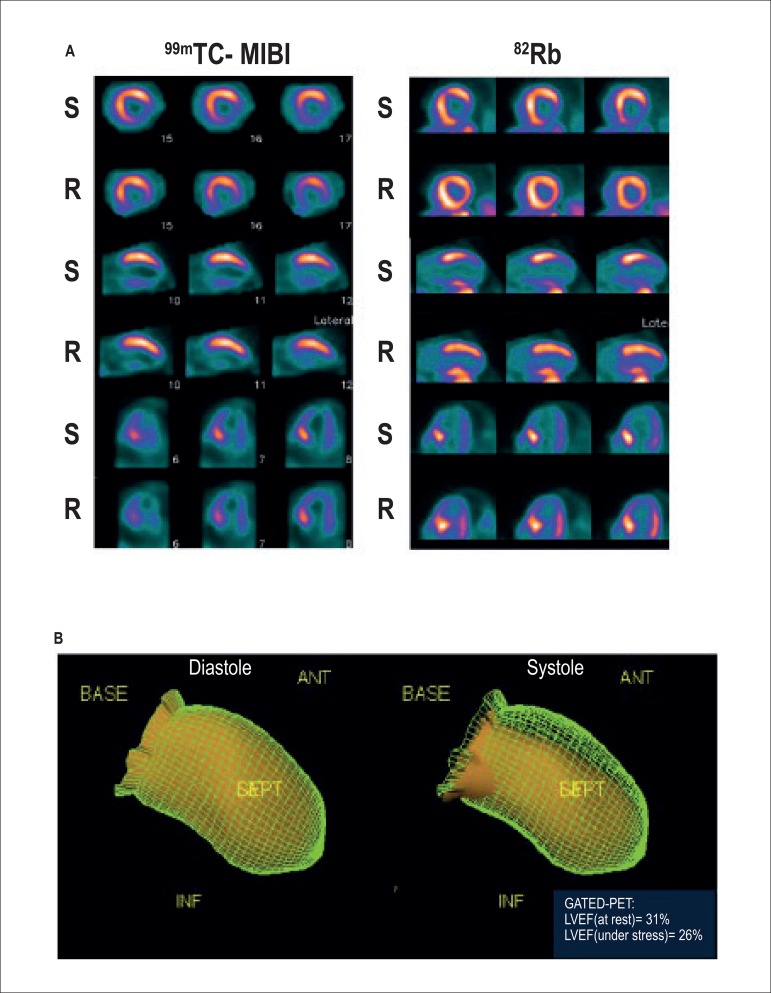
A) Myocardial perfusion at rest (R) and stress (S) with technetium-99m-Sestamibi (MIBI) to the right and rubidium-82 (82Rb) to the left. Ischemia can be observed in most prominent inferolateral wall in 82Rb. B) Left ventricular motility study (GATED-PET) shows apical akinesia and severe hypomotility of the left ventricular inferior and septal walls, with a decrease in ejection fraction under stress and the presence of transient ischemic dilation (volumetric ratio between stress and rest of 1.28).

We report the case of a patient with multivessel CAD referred for evaluation of myocardial perfusion, which was carried out through the two methods (Figure 1).

## Case Report

**Figure 2:**
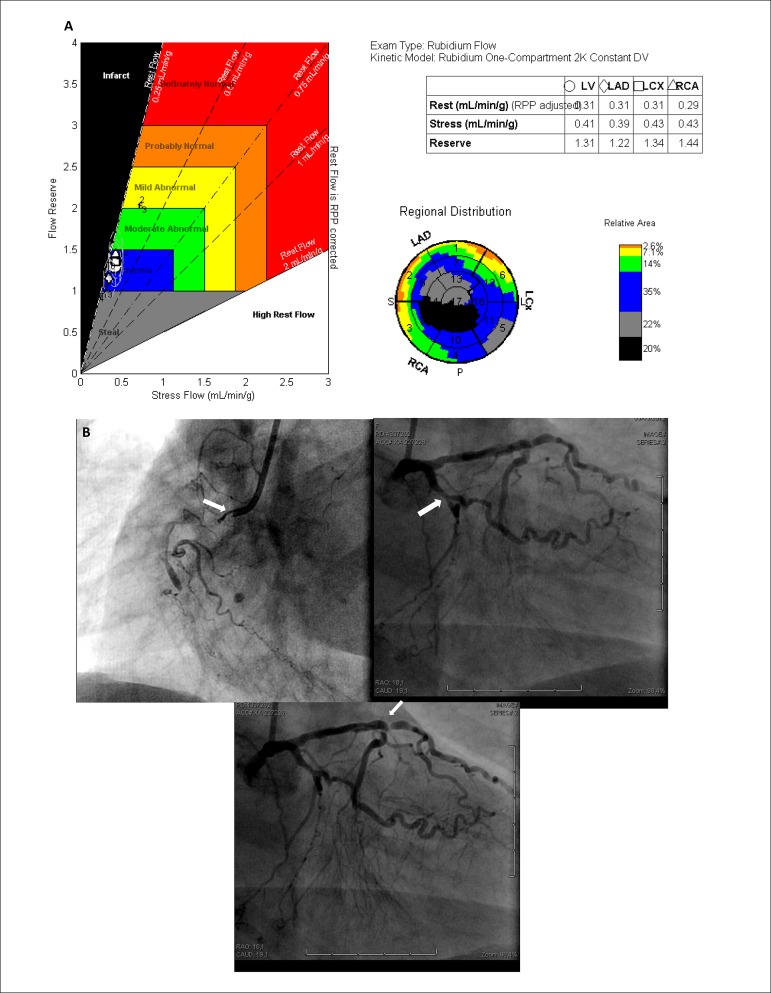
(A) Myocardial blood flow measurements (mL/ min/g) at rest and under stress, and coronary reserve in the territories of the left anterior descending (LAD), circumflex (LCX) and right coronary (RCA) arteries obtained with rubidium/positron-emission tomography. Observe the overall reduction in myocardial blood flow and left ventricular (LV) reserve and the territories of the three arteries (reserve <2.0). (B) Coronary angiography showing 100% occlusion in the anterior descending, circumflex and right coronary arteries, as well as the presence of grade 3 collateral circulation of multiple origin to the anterior descending artery, grade 2 in the right coronary artery and grade 3 in the second left marginal artery.

Female patient, 63 years old, reported chest burning pain and dyspnea on exertion for 2 years and was submitted to CAT, which detected CAD (Figure 2). She had hypertension, dyslipidemia, insulin resistance, heart failure and dilated cardiomyopathy to be clarified. On physical examination, the patient was in good general status, eupneic, acyanotic, regular heart rhythm, normal heart sounds with no murmurs, positive pulmonary breath sounds without adventitious sounds, unaltered abdomen and full pulses and with good amplitude, without edema, New York Heart Association (NYHA) functional class I. She was receiving carvedilol, losartan, spironolactone, furosemide, simvastatin, aspirin and clopidogrel. The resting echocardiogram showed significant degree of diffuse myocardial involvement; LV diastolic dysfunction grade 1, moderate-degree mitral regurgitation and ejection fraction of 30%. The baseline electrocardiogram showed inactive areas in the inferior and anterolateral walls, in addition to possible left ventricular overload.

The patient underwent myocardial perfusion scintigraphy with Sestamibi and rubidium-82 (^82^Rb), according to previously described protocol and technique.^1^ Initially, the resting image was carried out; approximately 2 hours later, the stress imaging was performed (Figures 1 and 2) using dipyridamole as a stress agent. The findings showed greater extension of perfusion alterations in the examination with ^82^Rb, in addition to coronary reserve alteration in all arterial territories.

Currently, the patient is being followed with optimal medical treatment due to the high risk and the presence of well-developed collateral circulation.

## Discussion

The established method for assessing perfusion and myocardial function, with an important role in risk stratification of patients with known or suspected CAD is cardiac SPECT with ^99m^Tc-Sestamibi. However, some disadvantages of the study related to the presence of image artifacts, the long duration of the examination and the possibility of underestimating ischemia severity in patients with multivessel disease ^2^ should be considered.

Among the noninvasive methods of LV perfusion and wall motion assessment, PET-CT with ^82^Rb has shown higher sensitivity and accuracy.^3^ This is a positron-emitting radionuclide that has characteristics similar to those of potassium and an ultrashort half-life of 75 seconds.

The advantages of performing tests with ^82^Rb in PET-CT are: better image quality due to attenuation correction, reduced examination time (approximately 40 minutes), less radiation exposure, and the possibility of quantification of myocardial blood flow and coronary flow reserve.^4,5^ Despite the high cost, this test allows the noninvasive evaluation of CAD, providing new data with probable impact on patient management^6^ and, eventually, can prevent costly interventions that do not result in clinical improvement.

The quantification of coronary flow reserve with ^82^Rb is calculated by dividing the blood flow under stress by that at rest, considering the coronary territories of the anterior descending, right coronary and circumflex arteries, as well as that of the LV as a whole. This index provides subsidies to differentiate patients with ischemia in the territory supplied by an artery with less severe stenosis from those with multivessel disease (balanced ischemia) because in these cases the reserve is globally decreased.^1,7^

In a recently published study, the coronary blood flow was considered an independent risk factor for symptomatic patients with normal myocardial perfusion study on PET.^8^ Other published studies have shown subclinical abnormalities in myocardial blood flow or coronary flow reserve in different cohorts of patients, including obese, diabetic, smoker, hypertensive and HIV-positive patients,^9,10^ with microcirculation disease and dilated hypertrophic cardiomyopathy, which seems to have implications for the prognosis of these patients.

In this present case, the myocardial perfusion scintigraphy with Sestamibi showed a pattern of transient relative myocardial perfusion with Sestamibi, which seems visually less extensive than that observed in the study with ^82^Rb. Additionally, the quantification of myocardial blood flow and coronary reserve showed alterations in the three arterial territories, characterizing a worse prognosis. If the patient had not been submitted to assessment with ^82^Rb, further evaluation would be needed for the feasibility study, due to the small degree of transient defect detected by the examination with Sestamibi. This would increase the time of examination and radiation dose received by the patient.

In our country, it is not possible to routinely perform myocardial perfusion imaging with PET-CT and ^82^Rb due to several factors, such as limited availability of PET-CT equipment and strontium/rubidium generator. However, the technique has great applicability in nuclear cardiology, either with ^82^Rb or ammonia, particularly in the increase of prognostic information provided by the test, such as in the case of coronary flow reserve.

## References

[1] Yoshinaga K, Klein R, Tamaki N (2010). Generator-produced rubidium-82 positron emission tomography
myocardial perfusion imaging-From basic aspects to clinical
applications. J Cardiol.

[2] Yoshinaga K, Katoh C, Manabe O, Klein R, Naya M, Sakakibara M (2011). Incremental diagnostic value of regional myocardial blood flow
quantification over relative perfusion imaging with generator-produced
rubidium-82 PET. Circ J.

[3] Sampson UK, Dorbala S, Limaye A, Kwong R, Di Carli MF (2007). Diagnostic accuracy of rubidium-82 myocardial perfusion imaging
with hybrid positron emission tomography/computed tomography in the
detection of coronary artery disease. J Am Coll Cardiol.

[4] Sampson U K, Dorbala S, Limaye A, Kwong R, Di Carli MF (2007). Diagnostic accuracy of rubidium-82 myocardial perfusion imaging
with hybrid positron emission tomography/computed tomography in the
detection of coronary artery disease. J Am Coll Cardiol.

[5] McMahon SR, Kikut J, Pinckney RG, Keating FK (2013). Feasibility of stress only rubidium-82 PET myocardial perfusion
imaging. J Nucl Cardiol.

[6] Ghotbi AA, Kjaer A, Hasbak P (2014). Review: comparison of PET rubidium-82 with conventional SPECT
myocardial perfusion imaging. Clin Physiol Funct Imaging.

[7] Gibbons RJ, Chareonthaitawee P (2009). Establishing the prognostic value of Rb-82 PET myocardial
perfusion imaging: a step in the right direction. JACC Cardiovasc Imaging.

[8] Naya M, Murthy VL, Taqueti VR, Foster CR, Klein J, Garber M (2014). Preserved coronary flow reserve effectively excludes high-risk
coronary artery disease on angiography. J Nucl Med.

[9] Kaufmann PA, Camici PG (2005). Myocardial blood flow measurement by PET: Technical aspects and
clinical applications. J Nucl Med.

[10] Schindler TH, Cardenas J, Prior JO, Facta AD, Kreissl MC, Zhang XL (2006). Relationship between increasing body weight, insulin resistance,
inflammation, adipocytokine leptin, and coronary circulatory
function. J Am Coll Cardiol.

